# MiR-654-3p Constrains Proliferation, Invasion, and Migration of Sinonasal Squamous Cell Carcinoma *via* CREB1/PSEN1 Regulatory Axis

**DOI:** 10.3389/fgene.2021.799933

**Published:** 2022-01-12

**Authors:** Xiao Cui, Ying Yang, Aihui Yan

**Affiliations:** ^1^ Department of Otorhinolaryngology Head and Neck Surgery, The First Hospital of China Medical University, Shenyang, China; ^2^ Department of Hematology, Shengjing Hospital of China Medical University, Shenyang, China

**Keywords:** MiR-654-3p, CREB1, PSEN1, sinonasal squamous cell carcinoma, malignant progression, epithelial–mesenchymal transition

## Abstract

**Background:** MiR-654-3p can repress malignant progression of cancer cells, whereas no relative reports were about its modulatory mechanism in sinonasal squamous cell carcinoma (SNSCC). This research committed to approaching modulatory effect of miR-654-3p on SNSCC cells.

**Methods:** Bioinformatics methods were utilized for analyzing interaction of miR-654-3p/cAMP-responsive element binding protein 1 (CREB1)/presenilin-1 (PSEN1). Expression levels of miR-654-3p, CREB1, and PSEN1 mRNA were assessed by quantitative real-time polymerase chain reaction. Western blot was completed for level assessment of CREB1, PSEN1, and epithelial–mesenchymal transition–related proteins. The targeted relationship between miR-654-3p and CREB1, or CREB1 and PSEN1 was authenticated *via* dual-luciferase assay and ChIP assay. A trail of experiments *in vitro* was used for detection of the effects of miR-654-3p/CREB1/PSEN1 axis on malignant progression of SNSCC cells.

**Results:** CREB1 as the downstream target mRNA of miR-654-3p could activate transcription of its downstream target gene PSEN1. Besides, miR-654-3p could target CREB1 to repress PSEN1 expression, thus restraining proliferation, migration, invasion, epithelial–mesenchymal transition, and hastening apoptosis of SNSCC cells.

**Conclusion:** MiR-654-3p as an antitumor gene targeted CREB1 to hamper malignant progression of SNSCC through miR-654-3p/CREB1/PSEN1 axis.

## Introduction

Sinonasal squamous cell carcinoma (SNSCC) is a malignancy derived from nasal sinuses, making up 65% of all cases of rhinocarcinoma (Al-Qurayshi et al., 2020). Despite relatively low morbidity of primary SNSCC (only takes up to 3%–5% of head and neck squamous cell carcinoma), its heterogeneity and variation in tissue are generally high, with unusual etiology (such as human papillomavirus, wood chips, leather debris); thus, research on its pathological mechanism, as well as diagnostic and therapeutic regimens, is warranted (Lewis, 2016). Analyses from perspectives of etiology, epidemiology, clinical features, and relatively genetic profiles yield out that SNSCC have markedly different features from other head and neck squamous cell carcinoma (cancer of pharynx, oral carcinoma), and thus, SNSCC can be regarded as a solitary carcinoma (Llorente et al., 2014). Main clinical symptoms of SNSCC conclude rhinocleisis, nosebleed, rhinorrhea, and facial pain; however, most patients present with advanced disease at diagnosis because this series of symptoms is inconspicuous in the early stage (Al-Qurayshi et al., 2020). Hence, a thorough inquiry of SNSCC is rather influential in the area of tumor research.

Existing studies exhibited that miR-654-3p exerts a tumor-repressive effect in varying cancers. For example, miR-654-3p hinders malignant progression of non–small cell lung cancer through PLK4 repression (Pu et al., 2020). MiR-654-3p hampers proliferative, migratory, and invasive potentials of hepatocellular carcinoma, and it is a potential prognostic biomarker (Yang et al., 2020). Besides, miR-654-3p downregulation betokens dismal outcomes of patients with colorectal cancer as well as facilitates malignant progression of the disease (Zhang et al., 2020). Nonetheless, previous studies of SNSCC failed to deal with miR-654-3p. To investigate the impact of miR-654-3p on occurrence and progression of SNSCC, this study focuses on modulatory effect of miR-654-3p on SNSCC.

cAMP-responsive element binding protein 1 (CREB1) as a member of CREB family binds to cAMP-responsive element, thus activating cAMP. Wilderness investigations manifested the involvement of CREB1 in multiplex signaling pathways relative to various cancer cells. For instance, a study demonstrated that imperatorin targets CREB1 to constrain transforming growth factor β 2/ERK axis, thus inhibiting metastasis of esophageal cancer (Xu et al., 2020). Hu et al. (2019), who looked at breast cancer, displayed that CREB1 exerts oncogenic effect through regulating CREB1/lin28/miR-638/VASP regulatory network. In addition, CREB1 is an oncogene in colon carcinoma (Han et al., 2020). Thus, CREB1 plays a vital role in modulating occurrence and progression of tumors. Nonetheless, the existing accounts fail to unveil the role and molecular mechanism of CREB1 in SNSCC, and therefore, we committed to unraveling this issue, as well as mechanism of tumorigenesis.

Dysregulation of presenilin-1 (PSEN1), the dominant component of *γ*-secretase complex, is a key regulator in tumorigenesis and progression, participating in biological and pathological processes of colorectal cancer, bladder cancer, liver cancer, and so on (Dabrowska et al., 2011; Deng et al., 2015; Ma et al., 2018). Current research denoted that PSEN1 is involved in multiple tumorigenesis, such as cell proliferation, migration, invasion, and apoptosis. PSEN1 hastens gastric cancer invasion and metastasis, which may be a possible biomarker and therapeutic target (Li et al., 2016). Besides, high expression of PSEN1, a tumor repressor, is implicated in the favorable prognosis of patients with type lumA breast cancer (Orzechowska et al., 2016). Numerous studies theorized that PSEN1 repression can hasten radiotherapy and chemotherapy resistance of varying cancers including esophageal cancer and bladder cancer (Deng et al., 2015; Meng et al., 2016). Nevertheless, earlier studies did not find molecular mechanisms by which PSEN1 modulates SNSCC progression. This study committed to unveiling upstream and downstream modulatory sites of PSEN1 in SNSCC progression and the possible impact of PSEN1 on SNSCC.

In the study, experimental results revealed that miR-654-3p hindered cell proliferation, migration, and invasion and stimulated cell apoptosis in SNSCC. Besides, miR-654-3p repressed cell malignant behaviors *via* targeting CREB1 in SNSCC. Meanwhile, it was ascertained that miR-654-3p downregulated PSEN1 through CREB1 suppression, thus constraining SNSCC cell malignant progression. Hence, miR-654-3p/CREB1/PSEN1 regulatory axis can serve as a novel target for SNSCC management.

## Materials and Methods

### Bioinformatics Analysis

Downstream target genes of miR-654-3p were predicted through mirDIP, Targetscan, miRDB, and starBase databases (http://ophid.utoronto.ca/mirDIP/; http://www.targetscan.org/mamm_31/; http://mirdb.org/; http://starbase.sysu.edu.cn/starbase2/). Venn diagram was plotted for intersection. String database (https://string-db.org/) was used for protein–protein interaction (PPI) network analysis on genes in the intersection; those that had the highest connectivity were selected as downstream target genes. Targetscan was implemented for prediction of binding sites between downstream target genes and miR-654-3p. The htfTarget, TRRUST, and ENCODE databases were introduced to predict downstream target genes of CREB1, and the intersection was selected by plotting Venn diagram. JASPAR (http://jaspar.genereg.net/) was utilized to predict the binding sites of transcription factor CREB1 and the target gene.

### Cell Culture

Human SNSCC cell line RPMI2650 (BNCC233970) was provided by BeNa Culture Collection. Cells were cultured in eagle minimal essential medium (ATCC, United States) with 10% fetal bovine serum (FBS; Thermo Fisher Scientific, United States) in an incubator (Thermo Fisher Scientific) at 37°C with 5% CO_2_.

### Cell Transfection

MiR-654-3p mimic and inhibitor, as well as corresponding negative controls (NCs), were provided by Shanghai GenePharma Co. Ltd., China. Complementary DNA (cDNA) sequences of CREB1 and PSEN1 were inserted into pcDNA3.1 (Honor Gene, China) to construct CREB1 overexpression plasmid (oe-CREB1) and PSEN1 overexpression plasmid (oe-PSEN1). Cells were seeded in triplicate in a 24-well plate and were transfected with 500 ng miR-654-3p mimic, inhibitor, NCs, oe-CREB1 and oe-PSEN1plasmids, and empty plasmid by using 2.5 µL Lipofectamine 2000 (Thermo Fisher Scientific). At 48 h after transfection, transfection efficiency was assayed by quantitative real-time polymerase chain reaction (qRT-PCR). Primer sequences were as follows: miR-654-3p mimic 5′-UGG​UUU​ACC​GUC​CAC​AUA​CAU-3′; mimic-NC 5′-GCU​GCU​GAA​UCA​UUA​UCC​CCU​U-3′. miR-654-3p inhibitor 5′-AAG​GUG​AUG​GUC​AGC​AGA​CAU​A-3′; NC-inhibitor 5′-AAG​UCA​GGU​GAU​GGA​CAG​CAU​A-3′.

### Flow Cytometry

In brief, 5 × 10^4^ cells were inoculated into 24-well plates and cultured in an incubator. Forty-eight hours later, cells were rinsed twice with phosphate-buffered saline (PBS). Annexin V and propidium iodide (BD Biosciences, United States) were recommended for double-staining. BD FACSCanto II (BD Biosciences) flow cytometer was utilized for analysis.

### Quantitative Real-Time Polymerase Chain Reaction

Trizol reagent (Thermo Fisher Scientific) was used to extract cell RNA. Hairpin-it miRNAs qRT-PCR kit (GenePharm) and PrimeScript RT Master Mix (Takara, China) were utilized for cDNA synthesis from miRNA and mRNA. SYBRA Green PCR Master Mix (Takara, Japan) was utilized for miRNA and mRNA expression detection. qPCR analysis was conducted on QuantStudio 3 (Thermo Fisher Scientific) PCR system per specifications (for primers, see Table 1). U6 and β-actin were taken as endogenous references for miR-654-3p and CREB1/PSEN1, respectively.

**TABLE 1:** Primer sequences in qRT-PCR.

Gene	Primer sequence (5′→3′)
miR-654-3p	F: TCG​GCA​GGU​GGU​GGG​CCG​CAG
	R: CAC​TCA​ACT​GGT​GTC​GTG​GA
CREB1	F: TGC​AAC​ATC​ATC​TGC​TCC​CA
	R: CTG​AAT​AAC​TGA​TGG​CTG​GGC
PSEN1	F: TAT​GGC​AGA​AGG​AGA​CCC​G
	R: CCA​TTC​CTC​ACT​GAA​CCC​G
U6	F: CTCGCTTCGGCAGCACA
	R: AAC​GCT​TCA​CGA​ATT​TGC​GT
β-Actin	F: TCC​GGC​ACT​ACC​GAG​TTA​TC
	R: GAT​CCG​GTG​TAG​CAG​ATC​GC

### Western Blot

First, radioimmunoprecipitation assay lysis buffer was utilized for cell lysis, and after 15 min of centrifugation at 12,000 revolutions/min, the total protein concentration was assessed with bicinchoninic acid/protein detection kit (Thermo Scientific, New York, United States); 25 μg proteins were separated using 12% sodium dodecyl sulfate–polyacrylamide gel electrophoresis. Afterward, proteins were transferred to polyvinylidene fluoride (Bio-Rad Laboratories, Inc., United States) membrane, which was sealed with 5% skimmed milk for 1 h at room temperature and cultured overnight with primary antibodies at 4°C. Antibodies were all bought from Abcam. Primary antibodies were all rabbit antibodies: anti-CREB1 antibody, anti-PSEN1 antibody, anti–β-actin antibody, anti–E-cadherin antibody, anti–N-cadherin antibody, and antivimentin antibody. Secondary antibody was goat anti-rabbit immunoglobulin G (IgG) H&L (HRP).

### Cell Counting Kit-8 Assay

RPMI2650 cells (1 × 10^4^ cells/well) were seeded into a 96-well plate. On days 0, 1, 2, 3, and 4 of incubation, 10 μL CCK-8 solution (MedChem Express, United States) was supplemented to the culture medium for another 3 h of incubation. Afterward, a microplate reader (Bio-Rad Laboratories, Inc.) was implemented for assessment of optical density value at 450 nm.

### Wound Healing Assay

RPMI2650 cells were seeded into 6-well plates (1 × 10^5^ cells/well). After cell confluence reached 80%, a 20 μL pipette tip was implemented to create scratches. Cells were then cultured for 24 h. Cell migratory potential was presented as a change in the width of scratch gap before and after healing. A microscope (XDS-800D; Shanghai Caikon Optical Instrument Co., Ltd., China) was used to observe images, and ImageJ software was utilized for analysis (relative wound width = wound width 24 h/wound width 0 h).

### Transwell Assay

First, 50 μL of Matrigel (BD Biosciences) was applied on the upper surface of 24-well plate Transwell inserts (BD Biosciences). Next, RPMI2650 cells (1 × 10^5^ cells/well) were resuspended in serum-free medium and inoculated into the upper chamber, while culture medium with 10% FBS (Thermo Fisher Scientific) filled the lower chamber. Twenty-four hours later, cells were subjected to 4% paraformaldehyde for cell fixing and 0.1% crystal violet for cell staining. Finally, a microscope was used for observation, and ImageJ software was used for analysis.

### Chromatin Immunoprecipitation

RPMI2650 cells were fixed with formaldehyde for 10 min to cross-linking of proteins with DNA, followed by fragmenting chromatin by an ultrasonic disruptor. After 10 min of centrifugation (4°C, 12,000*g*), the harvested cells were divided into two fractions for incubation overnight at 4°C with rabbit anti-IgG antibody (ab172730; Abcam, China) and CREB1 antibody (9,197; Cell Signaling, China), respectively. DNA–protein complex was precipitated with protein agarose/agarose, followed by centrifugation at 12,000*g* for 5°min, and the supernatant was discarded. Nonspecific complexes were eluted and decrosslinked overnight at 65°C. DNA fragments were then isolated and purified by phenol/chloroform. Primer sequences in qRT-PCR are presented in Table 2.

**TABLE 2:** ChIP-qPCR primer sequences.

PSEN1	Primer sequence (5′→ 3′)
Primer pair 1	F: AAC​AAG​GTT​GGC​AGT​GGG​TT
	R: CCA​CCC​CAA​GCT​ATC​CCT​TC
Primer pair 2	F: GAA​CAA​GGT​TGG​CAG​TGG​GT
	R: CCC​CAA​GCT​ATC​CCT​TCA​CA
Primer pair 3	F: ACA​AGG​TTG​GCA​GTG​GGT​TG
	R: ACC​CCA​AGC​TAT​CCC​TTC​AC

### Dual-Luciferase Reporter Gene Assay

First, pmirGLO–CREB1–3′-UTR wild type (WT) and pmirGLO–CREB1–3′-UTR-mutant type (MUT), pmirGLO–PSEN1-promoter-WT and pmirGLO–PSEN1-promoter-MUT luciferase reporter vectors (Promega, United States) were constructed. MiR-654-3p mimic/mimic-NC and pmirGLO–CREB1–3′-UTR-WT/pmirGLO–CREB1–3′-UTR-MUT, and oe-NC/oe-CREB1 and pmirGLO–PSEN1-promoter-WT/pmirGLO–PSEN1-promoter-MUT were cotransfected into RPMI2650 cells. After 48 h of cell culture, luciferase activity of each transfection group was assayed with luciferase activity assay kit (Promega) per kit specification.

### Statistical Analysis

GraphPad Prism 6 software (GraphPad Software, Inc., La Jolla, United States) was utilized for statistical analysis. All experiments were repeated in at least three replicates. The results were presented as mean ± standard deviation. *t* Test or one-way analysis of variance was performed for different comparisons. *p* < 0.05 means statistically significant differences. In figures, * represents *p* < 0.05.

## Results

### MiR-654-3p Is a Modulator of Proliferation, Migration, Invasion, and Apoptosis of SNSCC Cells

**FIGURE 1:**
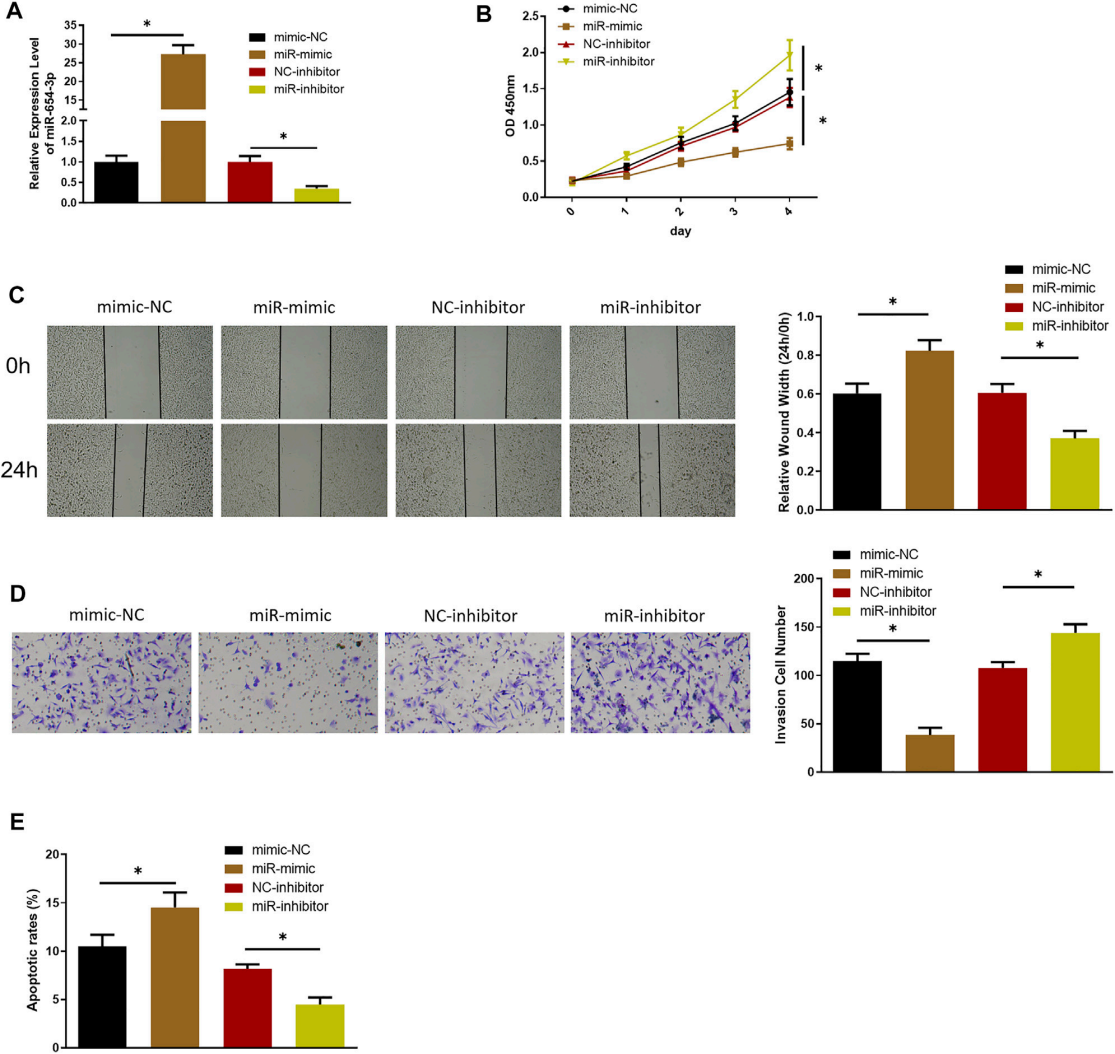
MiR-654-3p hinders proliferation, migration, and invasion and fosters apoptosis of SNSCC cells. **(A)** qRT-PCR assessed miR-654-3p level in each transfection group. **(B–D)** CCK-8, wound healing, Transwell assays measured proliferative, migratory, and invasive abilities of cells in each transfection group, respectively. **(E)** Flow cytometry detected cell apoptosis in each transfection group; *p < 0.05.

To investigate possible function of miR-654-3p in SNSCC, we overexpressed or silenced miR-654-3p by transfecting miR mimic or miR inhibitor into SNSCC cells. qRT-PCR was conducted to assess transfection efficacy (Figure 1A). To confirm the influence of miR-654-3p on proliferative potential of SNSCC cells, CCK-8 assay was conducted on each transfection group. As shown in Figure 1B, compared with the control group, proliferative capability of cells in miR-mimic group notably decreased, whereas that of cells in miR-inhibitor group had the opposite result. Wound healing and Transwell assays were carried out for cell detection of migratory and invasive capabilities. As presented in Figures 1C,D, these abilities of cells in miR-mimic group conspicuously decreased, whereas those in miR-inhibitor group showed an opposite trend. Finally, to affirm the impact of miR-654-3p on cell apoptosis, flow cytometry was implemented to assess apoptotic ratio of cells in each transfection group. The results manifested that apoptotic ratio of cells in miR-mimic group was the highest, whereas that of cells in miR-inhibitor group was the lowest, indicating the promotion of miR-654-3p overexpression on cancer cell apoptosis (Figure 1E). Together these findings illustrated that overexpressed miR-654-3p could hinder proliferation, migration, and invasion and foster apoptosis of SNSCC cells.

### MiR-654-3p Targets CREB1 in SNSCC Cells

**FIGURE 2:**
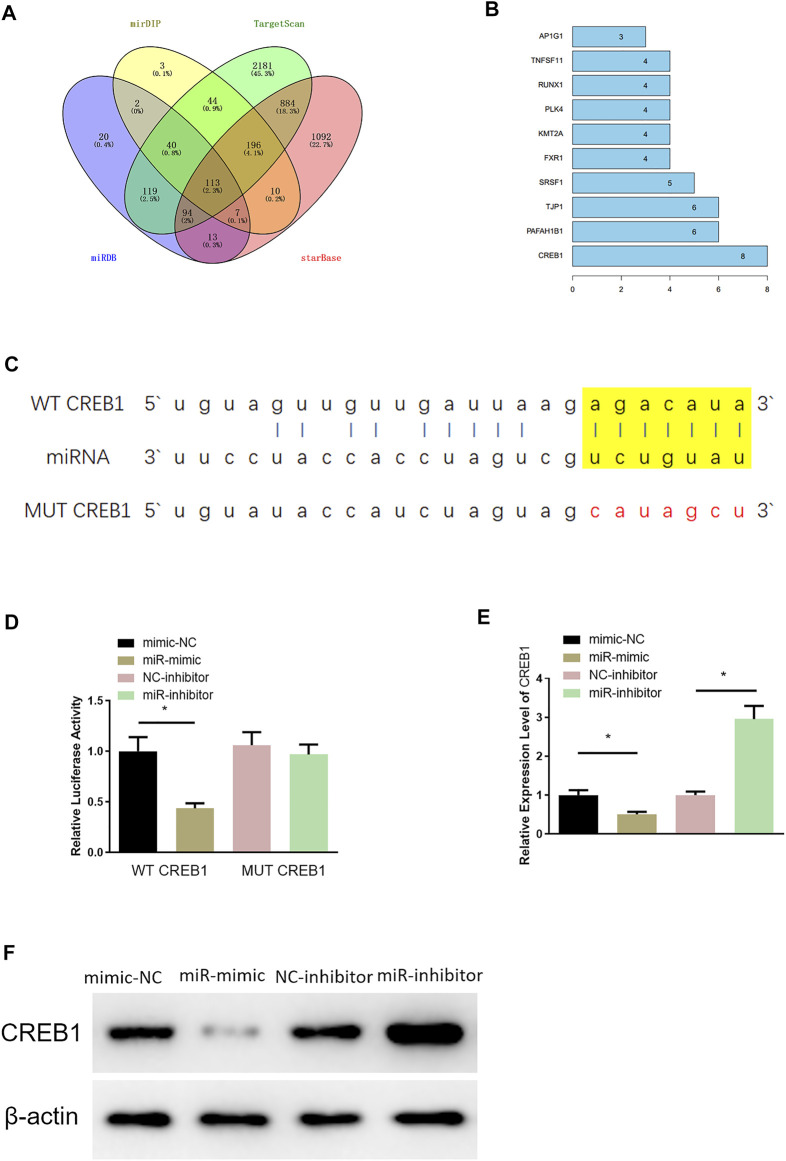
MiR-654-3p targets CREB1 in SNSCC cells. **(A)** Venn diagram of target genes predicted by miRDB, mirDIP, TargetScan, and starBase databases. **(B)** Genes of the Top10 highest connectivity in PPI network. **(C)** Targeted sites of miR-654-3p and CREB1 predicted by Targetscan. **(D)** The binding of miR-654-3p and CREB1 identified by dual-luciferase assay. **(E)** CREB1 mRNA level in each transfection group assessed by qRT-PCR. **(F)** CREB1 protein level in each transfection group measured *via* Western blot; *p < 0.05.

Through miRDB, mirDIP, TargetScan, and starBase databases, 113 potential downstream target genes of miR-654-3p were predicted (Figure 2A). On the basis, STRING database was utilized to construct PPI network and to identify a target gene with the highest connectivity, by which CREB1 was screened out as the downstream target gene of miR-654-3p (Figure 2B). Targetscan was used to predict specifically targeted sites of miR-654-3p and CREB1, and miR-654-3p was predicted to bind CREB1 (Figure 2C). Next, dual-luciferase assay was carried out to validate their binding relationship. As illustrated in Figure 2D, forced expression of miR-654-3p constrained luciferase activity of WT-CREB1 3′-UTR, whereas there were no notable changes in luciferase activity of MUT-CREB1 3′-UTR, indicating the binding relationship between miR-654-3p and CREB1. Afterward, qRT-PCR and Western blot were conducted to assess whether miR-654-3p affects CREB1 level, respectively. CREB1 mRNA and protein expression conspicuously decreased in miR-mimic group; in the opposite, CREB1 expression was dramatically increased in miR-inhibitor group (Figures 2E,F). Hence, it could be speculated that upregulated miR-654-3p downregulated CREB1 level in SNSCC cells.

### MiR-654-3p Is an Inhibitor in Malignant Progression of SNSCC *via* Targeting CREB1

**FIGURE 3:**
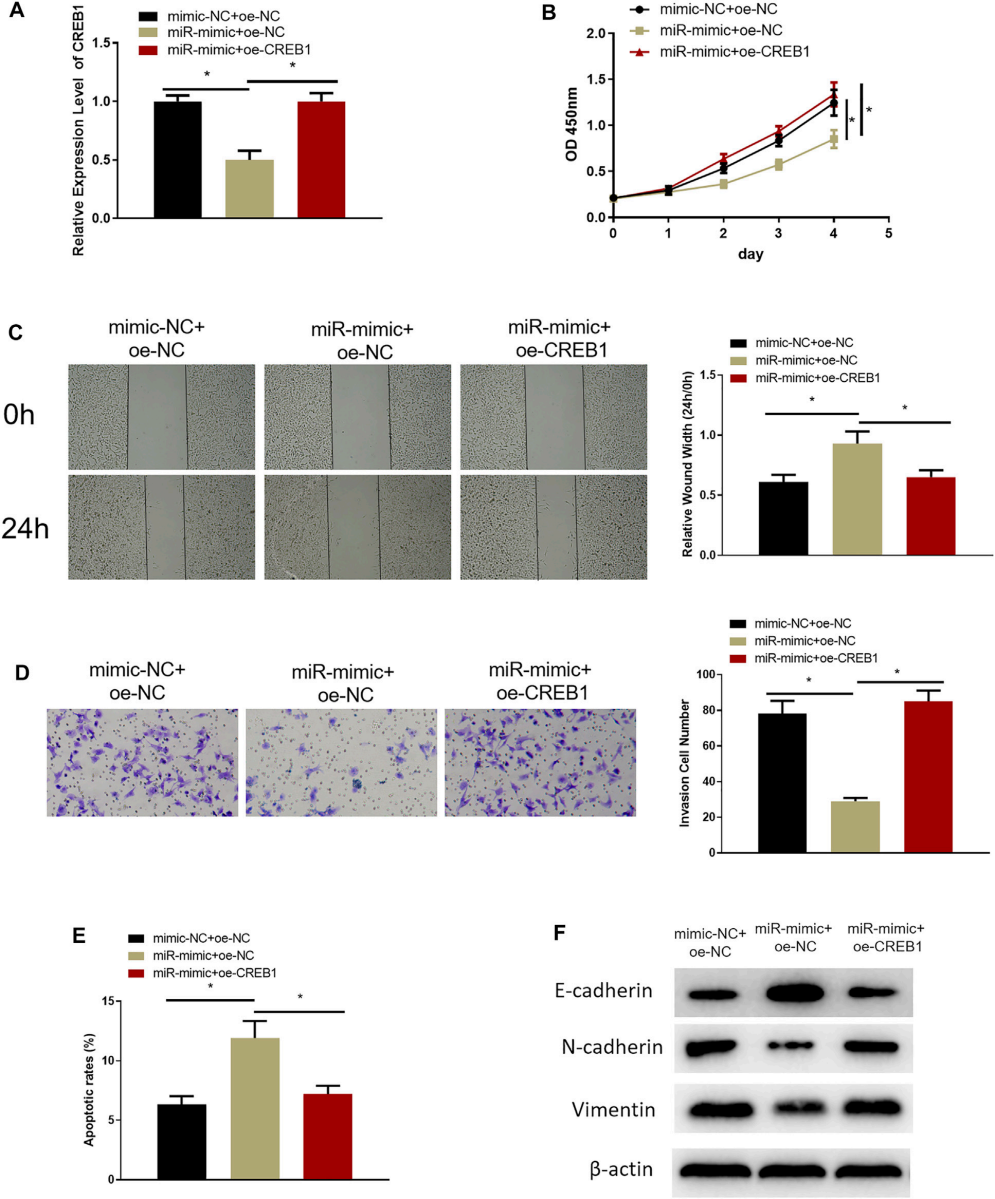
MiR-654-3p is an inhibitor in malignant progression of SNSCC *via* targeting CREB1. **(A)** Expression of CREB1 mRNA of cells in each transfection group assessed through qRT-PCR. **(B)** CCK-8 assay measured proliferative potential of cells in each transfection group. **(C)** Wound healing assay detected migratory ability of cells in each transfection group. **(D)** Transwell assessed invasive capability of cells in each transfection group. **(E)** Flow cytometry assessed cell apoptosis in each transfection group. **(F)** Western blot measured expression levels of EMT-related proteins in each transfection group; *p < 0.05.

Rescue experiments were designed to investigate the role of miR-654-3p/CREB1 regulatory axis on cell functional level. RPMI2650 cell line was utilized to establish miR-654-3p overexpression (miR-mimc + oe-NC) cell line, miR-654-3p and CREB1 overexpression (miR-mimic + oe-CREB1) cell line, and a control (NC-mimic + oe-NC) cell line for analysis of SNSCC cell biological functions. qRT-PCR, CCK-8, wound healing, and Transwell assays were conducted to assess CREB1 level, cell proliferative, migratory, and invasive capabilities of cells in each transfection group, respectively. qRT-PCR demonstrated that the decreased CREB1 level in miR-mimc + oe-NC group was reversed notably in miR-mimic + oe-CREB1 group (Figure 3A). Cell functional assays manifested that forced expression of miR-654-3p repressed proliferative, migratory, and invasive properties of SNSCC cells, whereas concurrent overexpressing miR-654-3p and CREB1 rescued these effects by miR-654-3p (Figures 3B–D). In addition, to evaluate the impact of miR-654-3p/CREB1 axis on apoptosis of SNSCC cells, flow cytometer was implemented for apoptotic detection of cells in each transfection group (Figure 3E). Compared with the NC-mimic + oe-NC group, the apoptotic ratio of cells in miR-mimc + oe-NC group was noticeably upregulated. But when compared with miR-mimc + oe-NC group, the apoptotic ratio of cells in miR-mimic + oe-CREB1 group was noticeably downregulated, demonstrating that the promoting effect of miR-654-3p on SNSCC cell apoptosis was reversed by CREB1. Moreover, numerous studies reported that CREB1 overexpression is associated with epithelial–mesenchymal transition (EMT) of cancer cells in a variety of cancers (Yan et al., 2018; Dong et al., 2019; Ma et al., 2019; Huang et al., 2020). On account of previous studies, we supposed that CREB1 may affect the EMT process of SNSCC cells; therefore, we carried out Western blotting to measure the expression levels of EMT-related proteins in each transfection group (Figure 3F). The results exhibited that miR-654-3p mimic repressed expression of stromal cell marker proteins N-cadherin and vimentin and increased expression of epithelial marker protein E-cadherin, but CREB1 could reverse this effect. To sum up, overexpressed miR-654-3p constrained proliferation, migration, invasion, and EMT process and facilitated cell apoptosis through targeting CREB1 in SNSCC cells.

### CREB1 Targets PSEN1 in SNSCC

**FIGURE 4:**
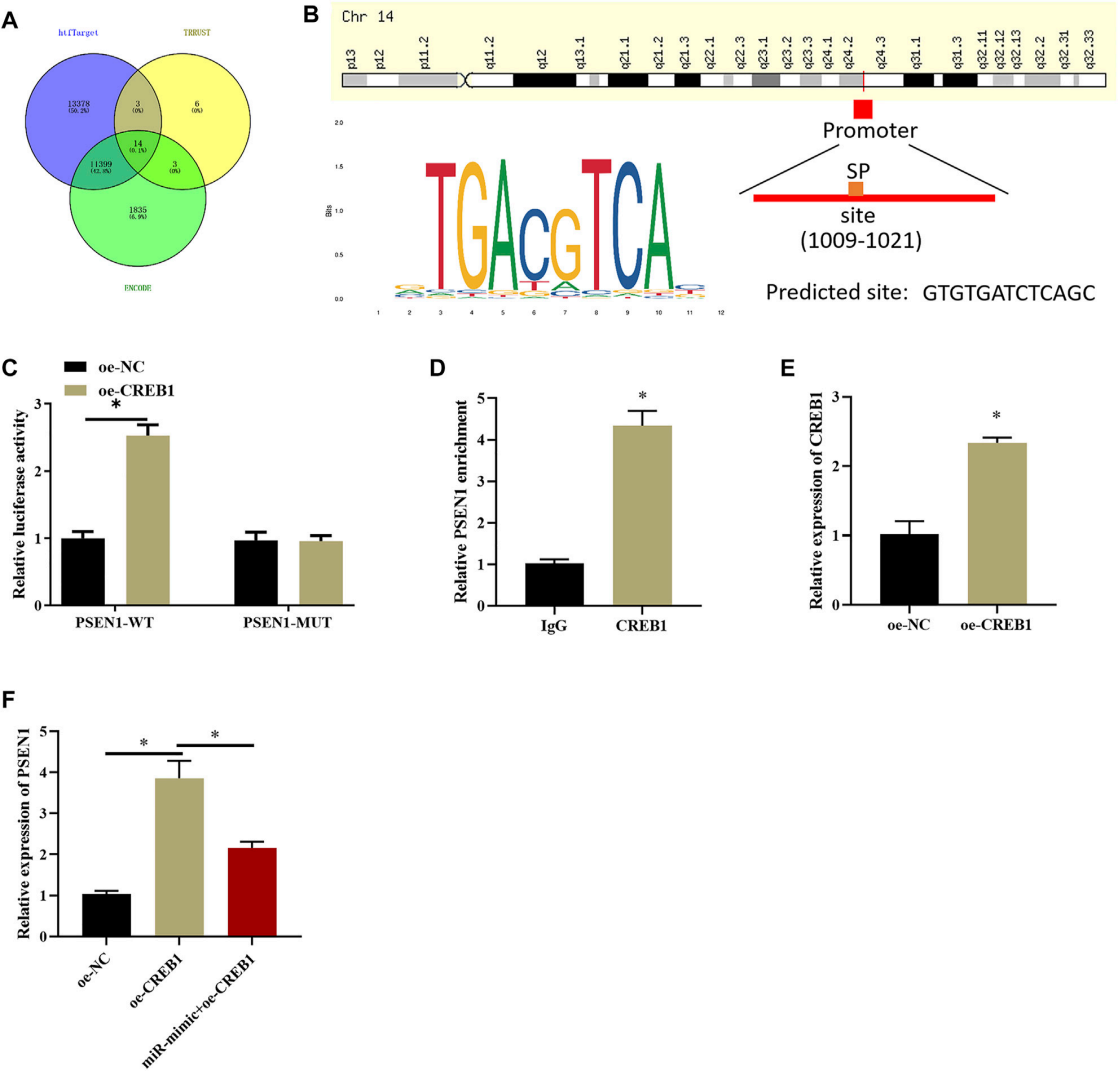
CREB1 increases PSEN1 level at transcription level. **(A)** Venn diagram plotted based on possible target genes downstream of CREB1 predicted by htfTarget, TRRUST, and ENCODE databases. **(B)** The motif map of CREB1 binding site in PSEN1 promoter region and CREB1 transcription factor. **(C)** Dual-luciferase assay verified binding of CREB1 and PSEN1. **(D)** ChIP detected that CREB1 can bind to promoter region of PSEN1. **(E)** qRT-PCR verified transfection efficiency of CREB1. **(F)** qRT-PCR assayed PSEN1 level after overexpressing CREB1 or miR-654-3p; *p < 0.05.

After clarifying the impact of CREB1 on SNSCC cells, target genes of the CREB1 transcription factor were identified by htfTarget, TRRUST, and ENCODE databases and were overlapped, and 14 possible target genes were obtained (Figure 4A). A study (Gou et al., 2020) proved that dysregulation of PSEN1 expression is implicated in dismal prognosis of patients with head and neck squamous cell carcinoma. Hence, PSEN1 was selected to study whether it also serves as a key regulator in SNSCC. First, we explored whether CREB1 can bind to PSEN1 promoter and increase its expression. Possible binding sites of PSEN1 in the promoter region of CREB1 were identified by bioinformatics methods (Figure 4B). Subsequently, dual-luciferase assay was introduced to validate binding relationship between CREB1 and PSEN1. As depicted in Figure 4C, forced expression of CREB1 could enhance luciferase activity of WT-PSEN1 3′-UTR, whereas no effect was observed in luciferase activity of MUT-PSEN1 3′-UTR, indicating that there was a binding relationship between CREB1 and PSEN1. ChIP results exhibited that enrichment of PSEN1 at the CREB1 binding site in SNSCC cells was markedly increased (Figure 4D). After oe-CREB1 and oe-NC transfection into RPMI2650 cells, transfection efficiency of CREB1 was assayed (Figure 4E). qRT-PCR results displayed that forced expression of CREB1 could notably enhance PSEN1 level, whereas concurrent overexpression of miR-654-3p and CREB1 notably decreased PSEN1 level (Figure 4F). Collectively, CREB1 could bind to the promoter region of PSEN1 and promote transcription of PSEN1. MiR-654-3p could target CREB1 to suppress PSEN1 level.

### MiR-654-3p Regulates PSEN1 *via* CREB1 to Hamper Malignant Progression of SNSCC Cells

**FIGURE 5:**
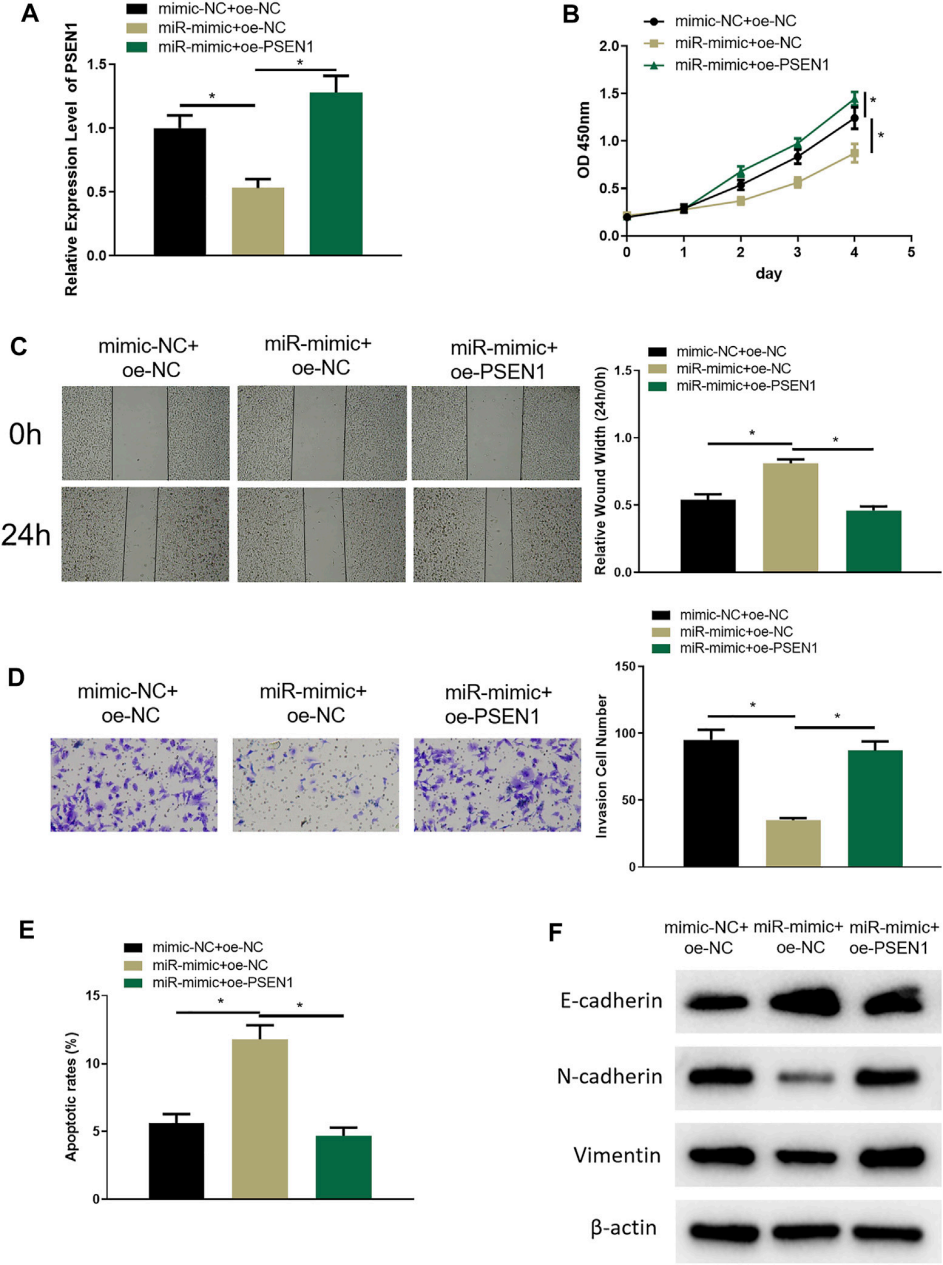
MiR-654-3p regulates PSEN1 *via* CREB1 to hamper malignant progression of SNSCC cells. **(A)** qRT-PCR assayed PSEN1 mRNA level in each transfection group. **(B)** CCK8 assayed cell proliferation in each group. **(C)** Wound healing assay assessed cell migratory property in each group. **(D)** Transwell assayed cell invasion in each group. **(E)** Flow cytometry assayed cell apoptosis in each group. **(F)** Western blot assayed EMT-related protein level in each group; *p < 0.05.

In the above sections, we clarified that miR-654-3p could regulate CREB1 to hinder the malignant behaviors of SNSCC cells, and CREB1 could increase PSEN1 expression. To investigate whether miR-654-3p can affect malignant progression of SNSCC cells by regulating PSEN1 through CREB1, RPMI2650 cell line was utilized to establish miR-654-3p overexpression (miR-mimc + oe-NC) cell line, miR-654–3p and PSEN1 concurrent overexpression (miR-mimic + oe-PSSEN1) cell line, and control cell line (NC-mimic + oe-NC) to study cell biological functions. qRT-PCR results denoted that PSEN1 mRNA expression was prominently reduced in miR-mimc + oe-NC group, and forced expression of PSEN1 could restore the expression (Figure 5A). Next, cellular functional experiments manifested that forced expression of miR-654-3p and PSEN1 concomitantly could reverse the repressive impact of overexpressing miR-654-3p alone on SNSCC cell behaviors (Figures 5B–D). Cell apoptosis in each group was assayed through flow cytometry, which disclosed that the promotion effect of miR-654-3p overexpression on SNSCC cells could be reversed by concomitant overexpression of miR-654-3p and PSEN1 (Figure 5E). EMT-related proteins were subjected to Western blot for expression analysis. As plotted in Figure 5F, compared with the control group, E-cadherin expression was notably increased, and N-cadherin and vimentin expression was conspicuously reduced in miR-mimc + oe-NC group, whereas those levels exhibited the opposite trend in miR-mimic + oe-PSEN1 group, demonstrating that miR-654-3p could repress EMT process in SNSCC cells, whereas this effect could be reversed by forced expression of PSEN1. Hence, miR-654-3p could regulate PSEN1 *via* CREB1 to hamper malignant progression of SNSCC cells.

## Discussion

SNSCC is the most common rhinocarcinoma, whereas relative basic research and diagnostic and therapeutic avenues are scarce. The incidence of SNSCC is low compared with other cancers; thus, it is hard to obtain pathological specimens, which leads to less research in SNSCC than other cancers (Lechner et al., 2020). Despite low morbidity, SNSCC has high heterogeneity, which creates hardships for accurate diagnosis and therapeutic regimen establishment in clinical. Current studies evinced that miRNAs can influence onset and progression of SNSCC (Zhao and Wang, 2018; Meng et al., 2020). Hence, this study researched SNSCC pathogenesis from the perspective of miRNAs.

Existing reports manifested that miR-654-3p is a key miRNA that exerts an antitumor role (Pu et al., 2020; Zhang et al., 2020). Duan et al. (2020) disclosed that miR-654-3p hinders cell proliferation, invasion, and sphere formation in breast cancer. Xiong and others manifested that miR-654-3p constrains cell viability and hastens cell apoptosis *via* targeting RASAL2 in non–small cell lung cancer (Xiong et al., 2021). But previous evidence rarely reported the modulatory role of miR-654-3p in SNSCC. In agreement with earlier studies, our research displayed that miR-654-3p could repress cell malignant behaviors in SNSCC.

In this study, we manifested by bioinformatics methods that CREB1 was a target gene downstream of miR-654-3p, which was proven to facilitate varying malignancies including glioma as a proto-oncogene transcription factor (Chen et al., 2017). Ye et al. (2017) disclosed that CREB1 is markedly increased in colorectal cancer and plays as an oncogene. Li et al. (2020) reported similar findings that forced expression of CREB1 reversed repressive effect of miR-383 on colorectal cancer cell proliferation and glycolysis, as well as promotion effect on cell apoptosis. Moreover, CREB1 silence hinders cell proliferation and EMT in prostate cancer (Wang et al., 2017). As depicted by previous studies, miR-654-3p could decrease the CREB1 level, thus constraining promotion effect of CREB1 on proliferation and EMT in SNSCC.

CREB, as a transcription factor, can bind to CRE sequence and modulate gene transcription, thus upregulating or downregulating specific gene expression (Cha-Molstad et al., 2004). In this study, we revealed that PSEN1 was a target gene downstream of CREB1, and CREB1 fosters PSEN1 transcription through bioinformatics analysis. PSEN1, mainly located in the endoplasmic reticulum, is a ubiquitously expressed protein with multiple transmembrane domains (Li et al., 2016). Earlier studies denoted that miR-193a downregulates the PSEN1 level to constrain cell proliferation and invasion of gastric cancer (Pan et al., 2021), which was in accord with our results. We confirmed through cellular experiments and rescue experiments that miR-654-3p modulated CREB1/PSEN1 axis to constrain proliferation, migration, and invasion and induce apoptosis of SNSCC cells. Besides, PSEN1/γ-secretase complex is conducive to the processing of P-cadherin, N-cadherin, and E-cadherin, thus modulating cell movement and invasion (Li et al., 2016). The result that PSEN1 fosters cancer cell EMT was also confirmed in gastric cancer and lung adenocarcinoma (Guo et al., 2020; Pan et al., 2021). Hence, we speculated that PSEN1 could modulate SNSCC cell EMT and verified the promotion effect by assessing expression of EMT-related proteins in multiple cotransfection groups, whereas miR-654-3p could modulate PSEN1 to hamper SNSCC cell EMT, which was in accord with earlier studies. These findings provide molecular mechanism by which miR-654-3p functions on SNSCC, which lay groundwork of miR-654-3p as a tumor repressor and contribute to precise treatment for SNSCC.

In summary, this study verified that miR-654-3p could target CREB1 to decrease PSEN1 level, thus hindering SNSCC cell proliferation, invasion, migration, and EMT and inducing cell apoptosis. Our research sufficiently proofed the connection between miR-654-3p expression and SNSCC malignant progression. Nevertheless, this study is deficient. The main weakness is the failure in identifying signaling pathways that miR-654-3p/CREB1/PSEN1 axis participates. Further work is required to investigate relative signaling pathways.

## Data Availability

The original contributions presented in the study are included in the article/Supplementary Material, further inquiries can be directed to the corresponding author.
