# Supplementary figures and images for: MiR-654-3p Constrains Proliferation, Invasion, and Migration of Sinonasal Squamous Cell Carcinoma via CREB1/PSEN1 Regulatory Axis
Source: Front Genet. 2022 Jan 12;12:799933. doi: 10.3389/fgene.2021.799933 (PMC8791623; doi:10.3389/fgene.2021.799933)

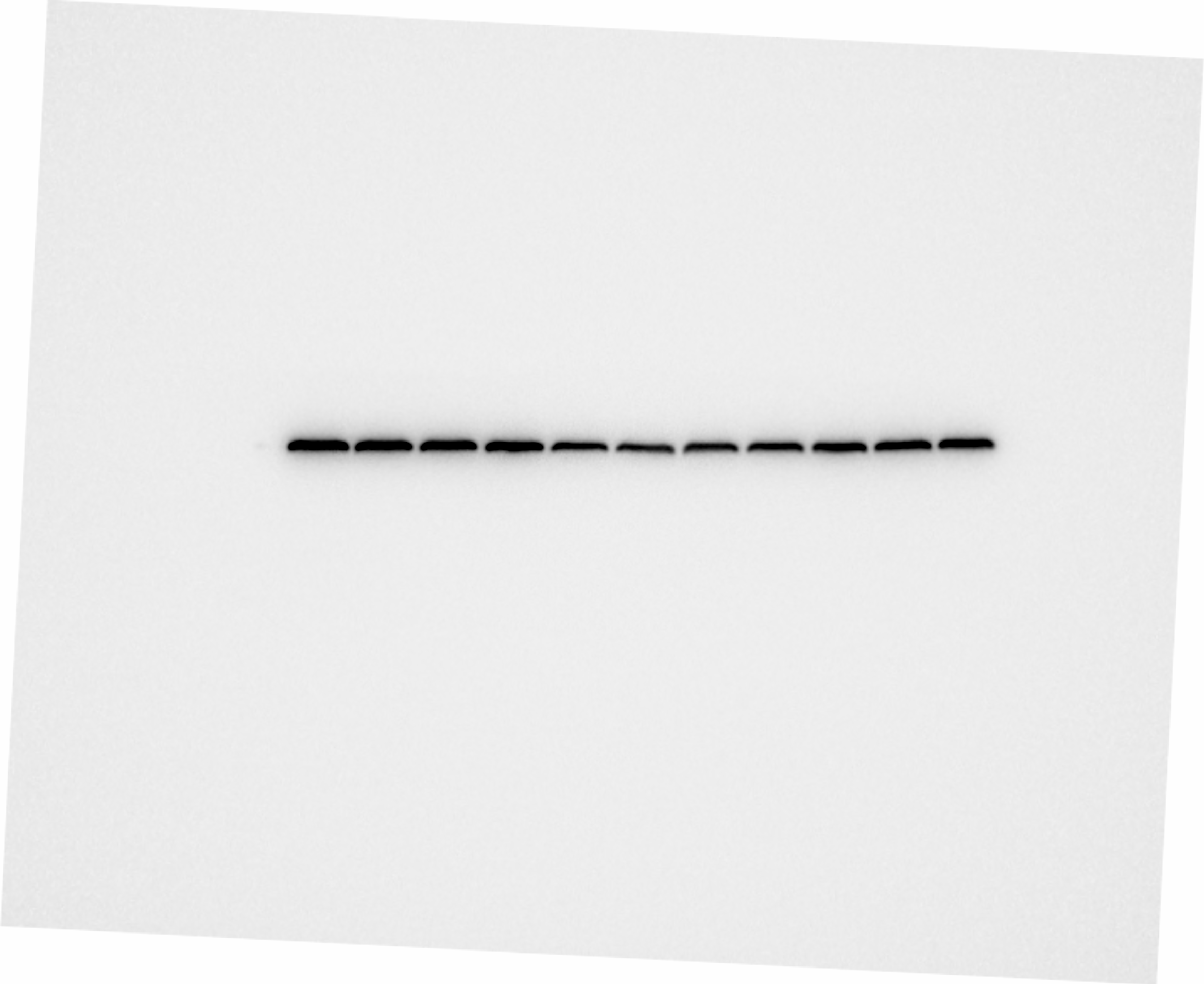

Supplement: Supplementary file 1 [file Image6.TIF]

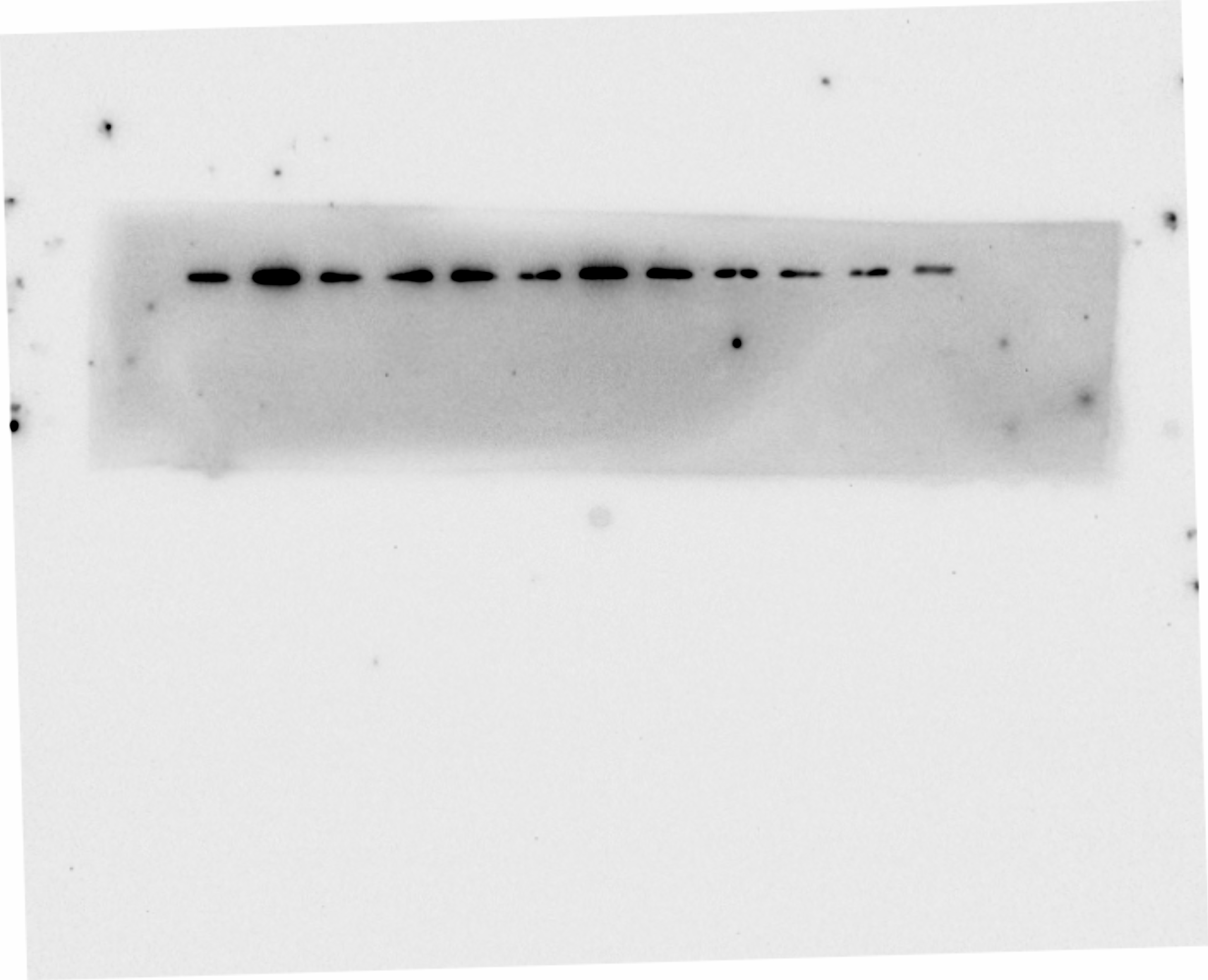

Supplement: Supplementary file 2 [file Image3.TIF]

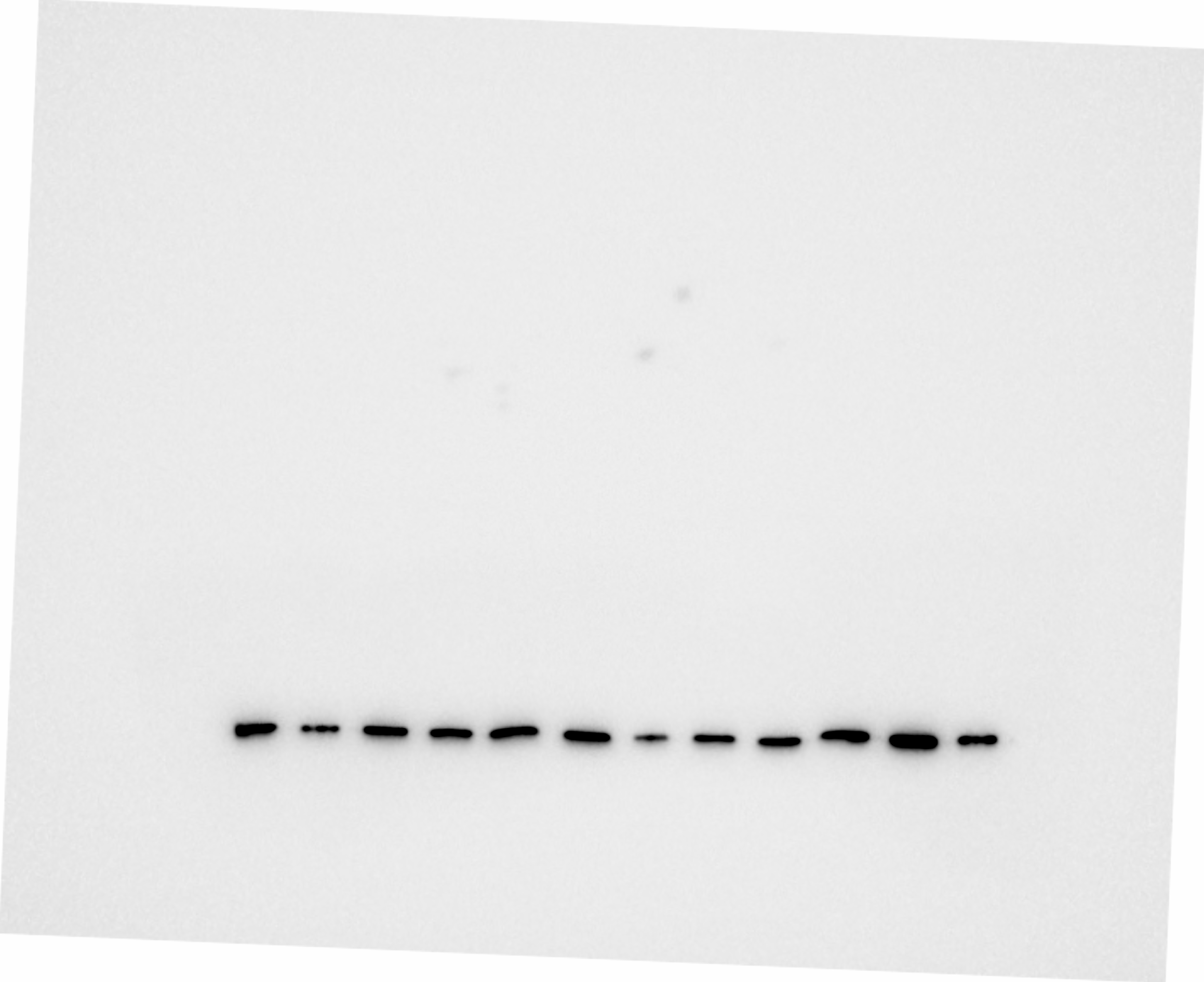

Supplement: Supplementary file 3 [file Image4.TIF]

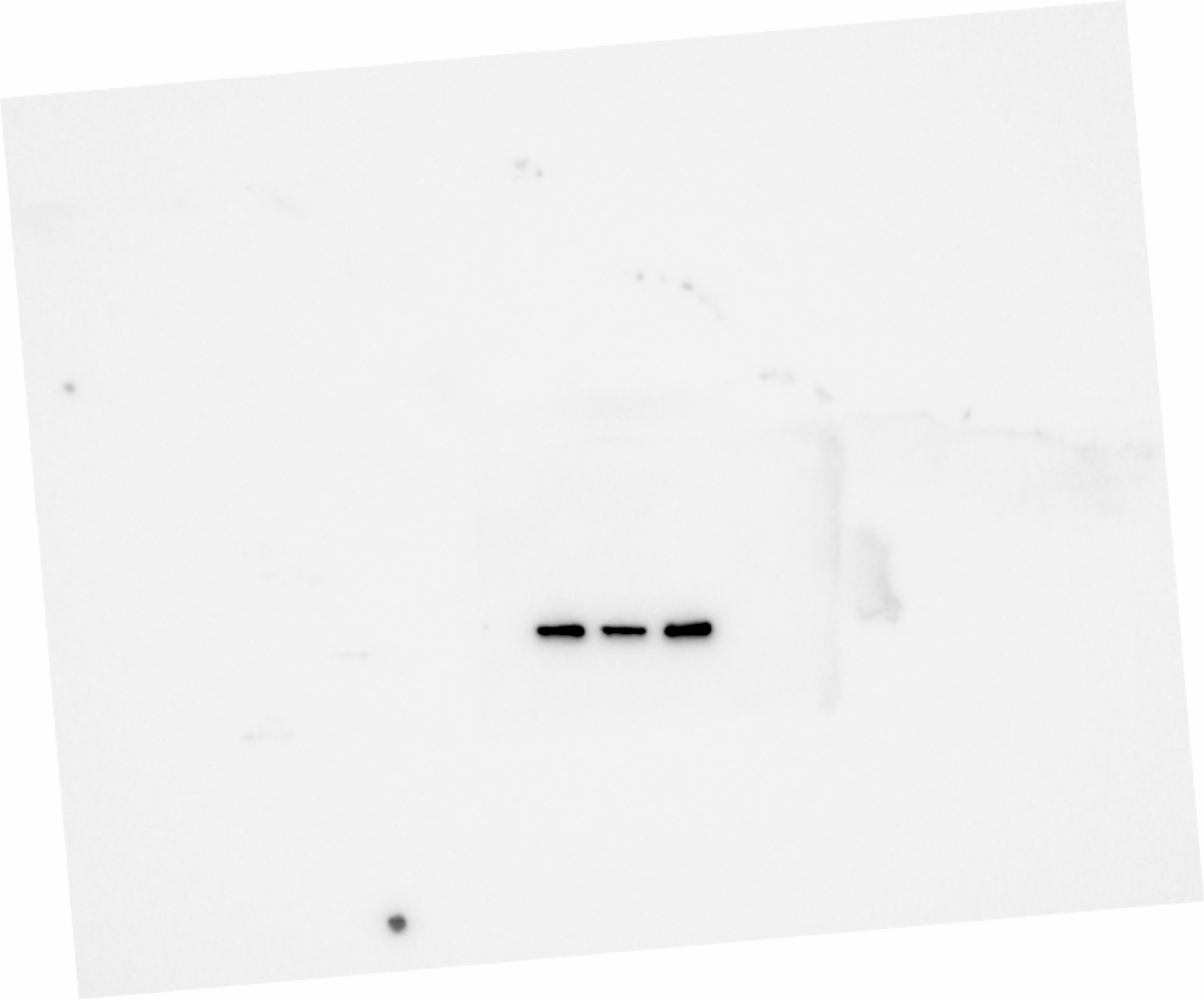

Supplement: Supplementary file 4 [file Image9.TIF]

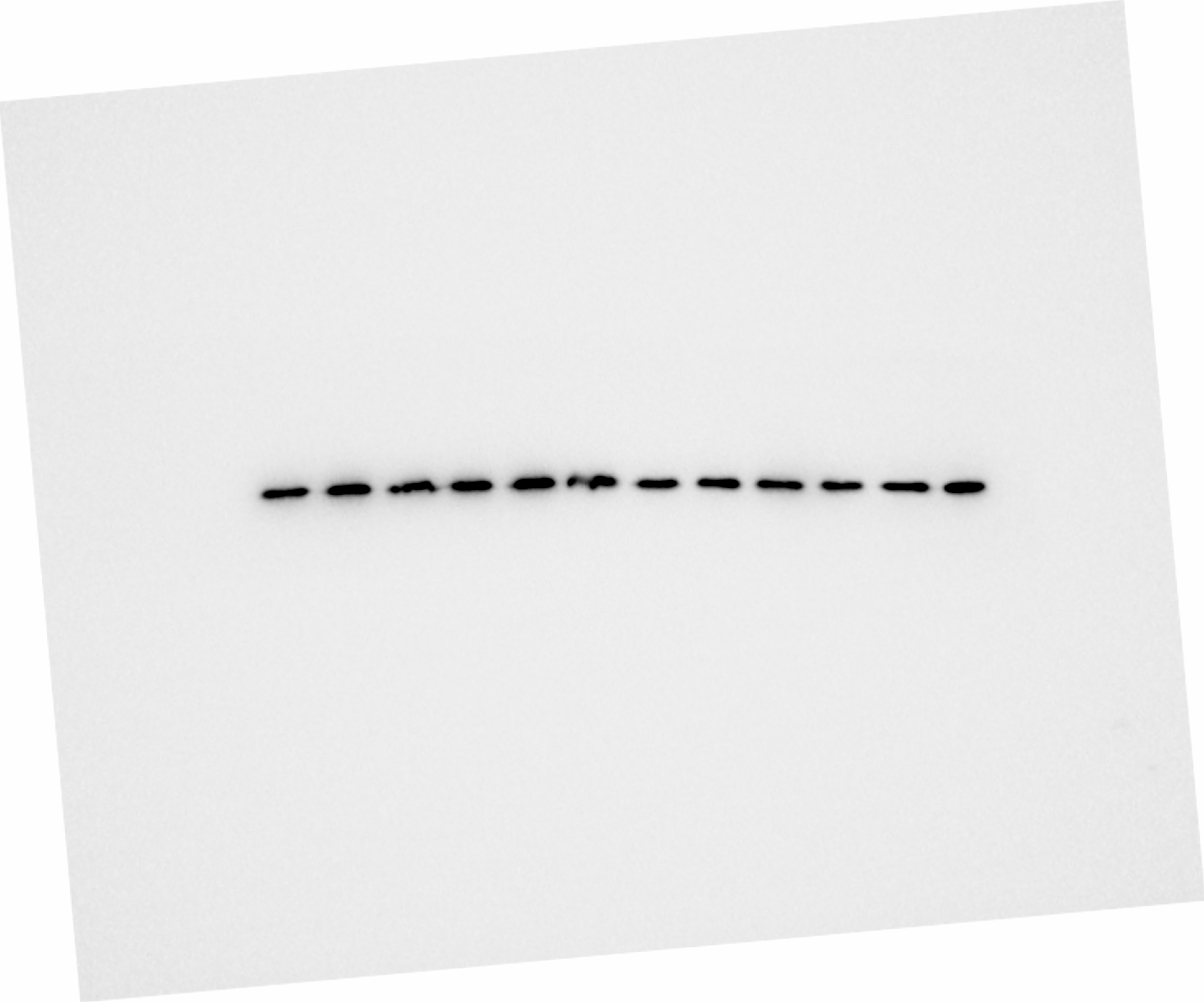

Supplement: Supplementary file 5 [file Image2.TIF]

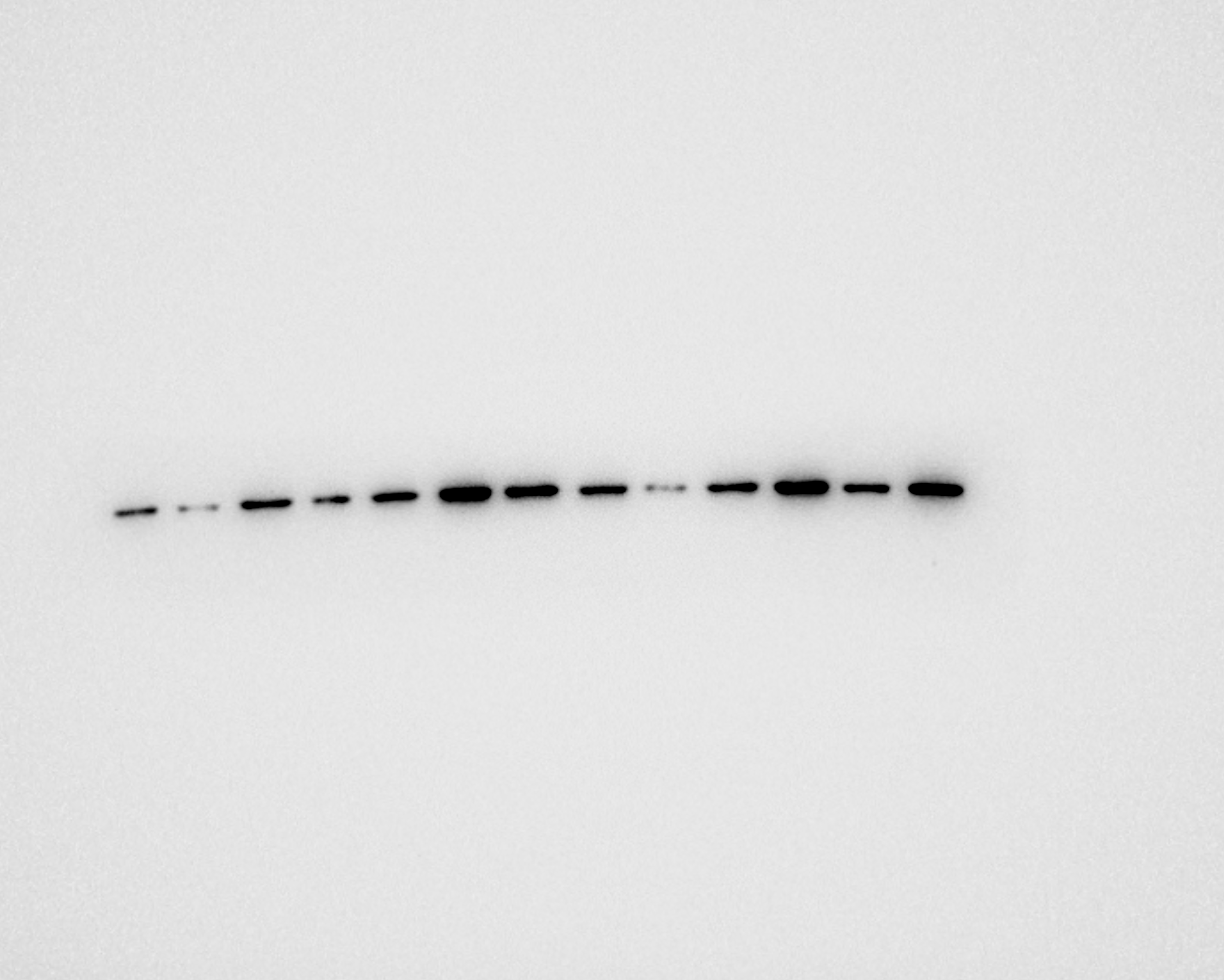

Supplement: Supplementary file 6 [file Image1.TIF]

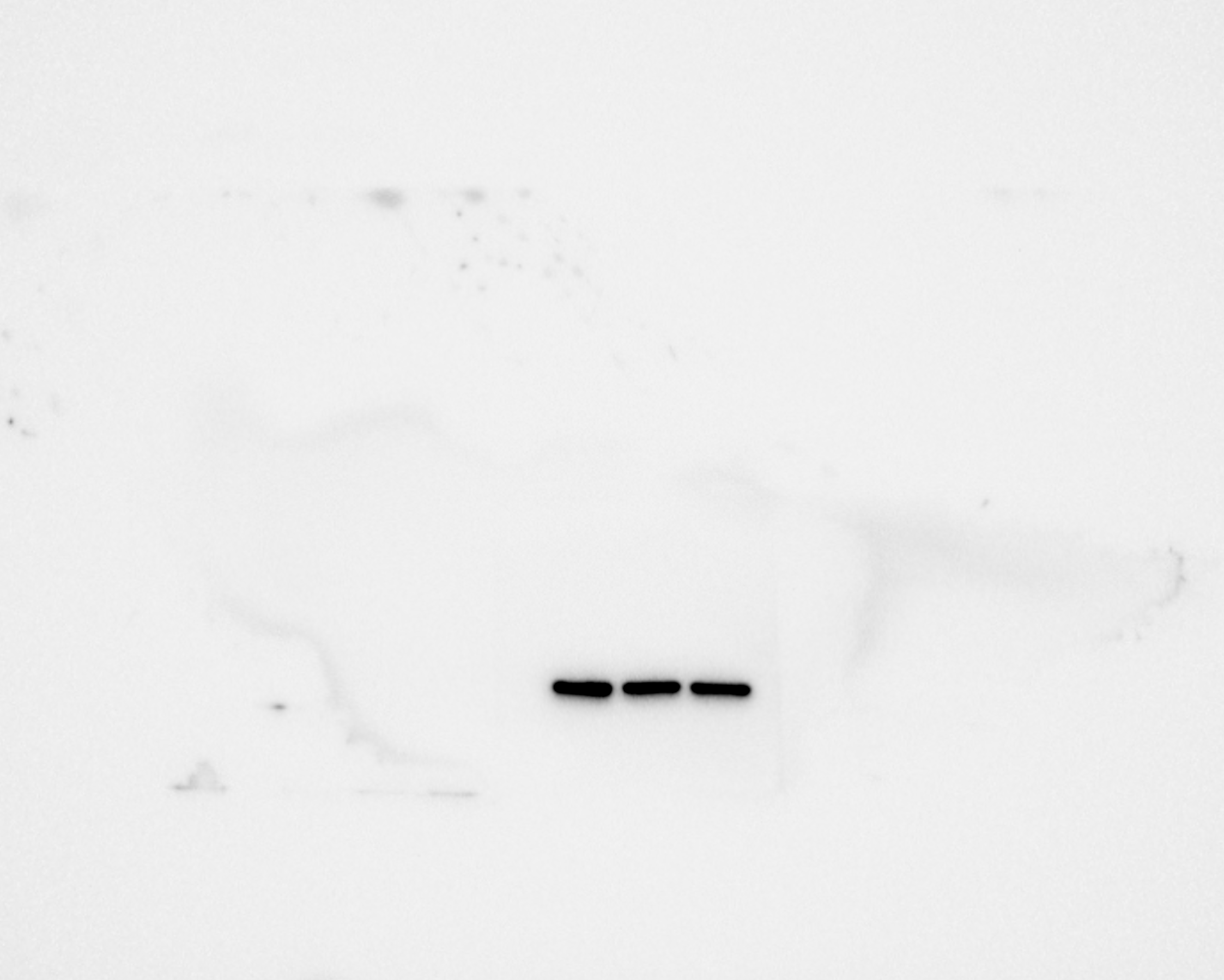

Supplement: Supplementary file 7 [file Image10.TIF]

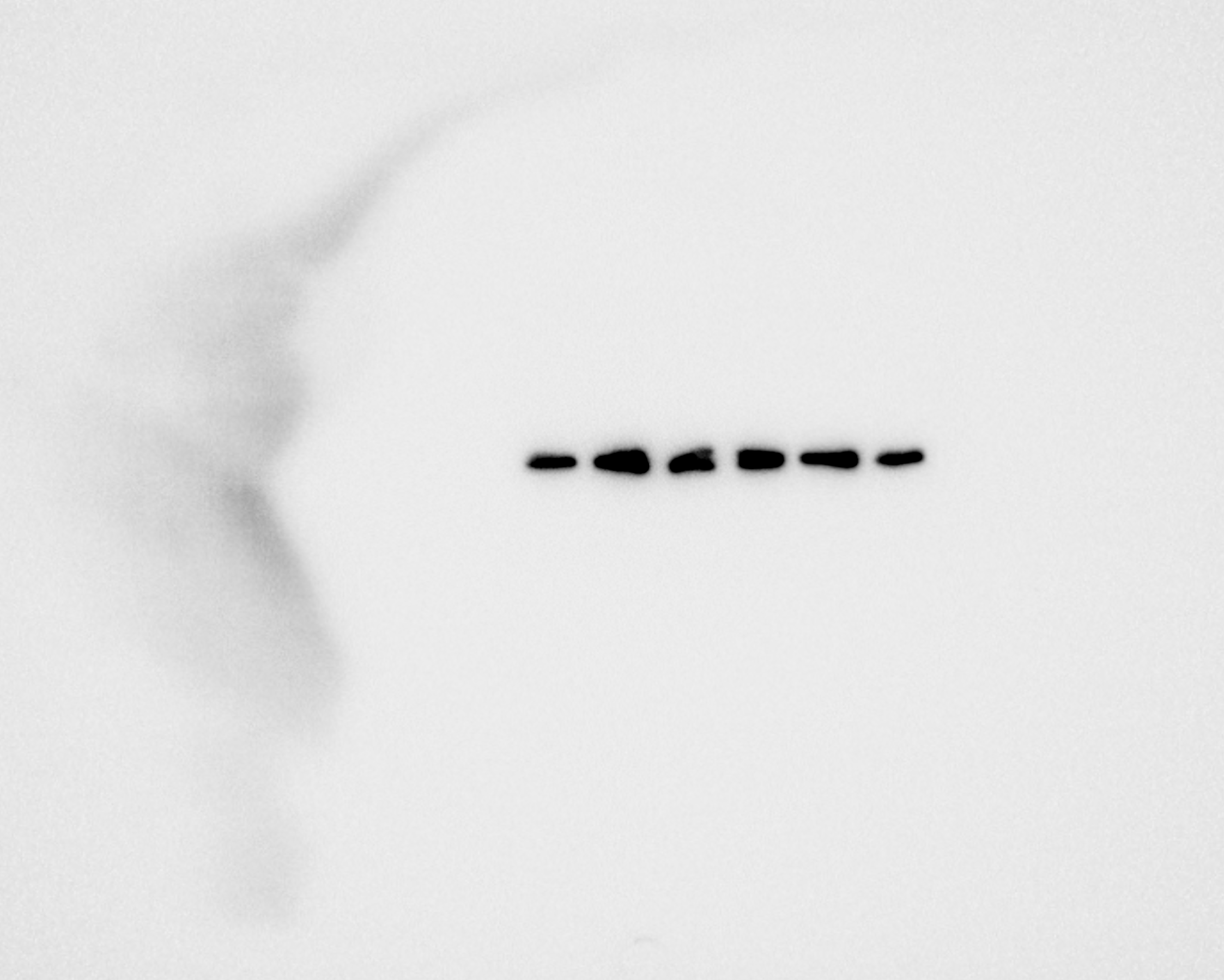

Supplement: Supplementary file 8 [file Image7.TIF]

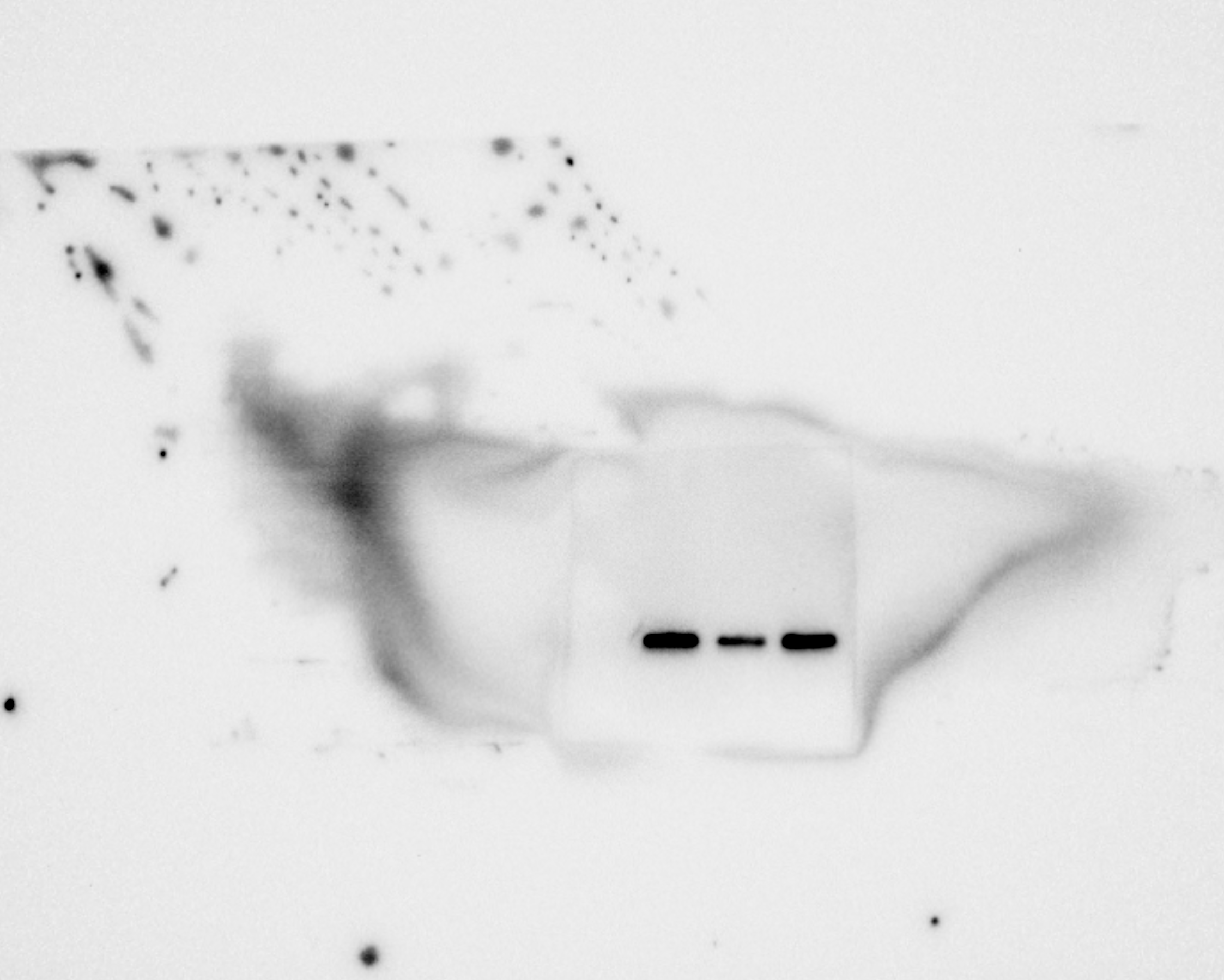

Supplement: Supplementary file 10 [file Image8.TIF]

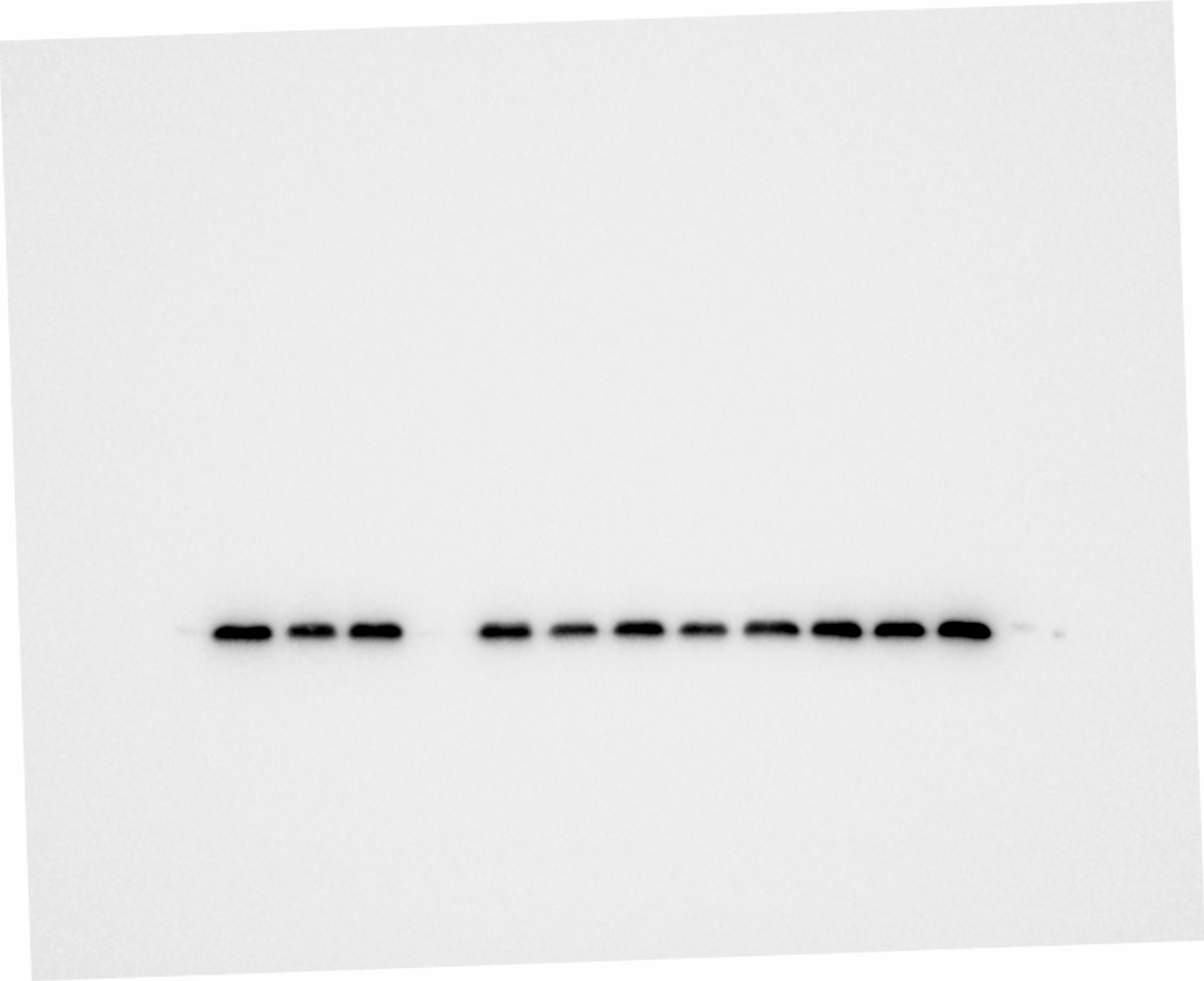

Supplement: Supplementary file 11 [file Image5.TIF]
